# Influence of Cyclodextrins on Thermosensitive and Fluorescent Properties of Pyrenyl-Containing PDMAA

**DOI:** 10.3390/polym11101569

**Published:** 2019-09-26

**Authors:** Qiujing Dong, Changrui Sun, Fangyuan Chen, Zheng Yang, Ruiqian Li, Chang Wang, Chunhua Luo

**Affiliations:** 1 School of Chemistry and Materials Engineering, Fuyang Normal University, Fuyang 236037, China; dongqj1980@163.com (Q.D.); sunchangrui1208@163.com (C.S.); cfy05038861@163.com (F.C.); 13955664328@163.com (Z.Y.); liruiqian2008@163.com (R.L.); bigceleron@163.com (C.W.); 2 Anhui Provincial Key Laboratory for Degradation and Monitoring of the Pollution of the Environment, Fuyang 236037, China; 3 State Key Laboratory of Molecular Engineering of Polymers (Fudan University), Shanghai 200433, China

**Keywords:** poly(*N*,*N*-dimethylacrylamide), cyclodextrins, pyrene, thermosensitive polymers, fluorescence

## Abstract

A series of pyrenyl-containing PDMAA copolymers were prepared by free radical copolymerization of dimethylacrylamide (DMAA) with pyrenebutanoyloxy ethyl methacrylate (PyBEMA). The structure of as-prepared copolymers was characterized by UV, FT-IR and ^1^H NMR spectroscopy. The effect of cyclodextrins (α-CD, β-CD and γ-CD) on the thermosensitivity and fluorescence of the copolymers in aqueous solutions were investigated. It was found that the as-prepared copolymers exhibit lower critical solution temperature (LCST)-type thermosensitivity. Cloud point (T_cp_) decreases with the increasing molar content of PyBEMA unit in the copolymers. T_cp_ of the copolymers increases after the CD is added from half molar to equivalent amount relative to pyrenyl moiety, and that further adding twice equivalent CD results in a slight decrease in T_cp_. The copolymers exhibit a pyrene emission located at 377 nm and a broad excimer emission centered at 470 nm. The copolymers in water present a stronger excimer emission (Intensity I_E_) relative to monomer emission (Intensity I_M_) than that in ethanol. The I_E_/I_M_ values decrease after the addition of equivalent α-CD, β-CD and γ-CD into the copolymers in aqueous solution, respectively. The I_E_/I_M_ values abruptly increase as the copolymers’ concentration is over 0.2 mg/L whether in ethanol solution or aqueous solution with or without CD, from which can probably be inferred that intra-polymeric pyrene aggregates dominate for solution concentration below 0.2 mg/L and inter-polymeric pyrene aggregates dominate over 0.2 mg/L. Furthermore, the formation of the CD pseudopolyrotaxanes makes it possible to form pyrene aggregates. For high concentration of 5 g/L, the copolymers and their inclusion complexes completely exhibit an excimer emission. The I_E_ values abruptly increased as the temperature went up to T_cp_, which indicates that the I_E_ values can be used to research phase separation of polymers.

## 1. Introduction

Stimuli-responsive fluorescent polymers have attracted enormous attention for their extensive applications in the fields of sensors, energy conversion, diagnostics, biolabeling, intracellular thermometers, and live cell imaging [1,2,3,4,5,6,7,8,9,10,11]. For the introduction of fluorescent dyes into stimuli-responsive polymers, many approaches can be applied to obtain stimuli-responsive fluorescent polymers, such as postmodification of polymer chains with a dye and direct (co)polymerization of fluorescent dyes by conventional, radical or RAFT (reversible addition-fragmentation chain transfer), or initiator with fluorescent dyes moiety [12,13,14,15,16,17,18,19,20,21,22,23,24,25,26,27,28]. Among them, the direct (co)polymerization of fluorescent monomers has been widely used due to its superiority in terms of consuming less time and incurring lower costs compared with other methods. Most stimuli-responsive fluorescent polymers are based on phase transition of polymers in solution exhibiting a lower critical solution temperature (LCST) or an upper critical solution temperature (UCST). Among the various classes of stimuli-responsive fluorescent polymers, dye-functionalized poly(*N*-isopropylacrylamide) (PNIPAM) is the most widely studied LCST-type polymer [29,30]. The pyrene is one of the most studied fluorescent dyes in chemistry. The heat-induced phase transition in water of a pyrene functionalized PNIPAM was first investigated by Winnik and co-workers who found that the pyrene excimer emission is affected by the phase transition [12,13]. Furthermore, pyrene is also used as a fluorescent probe to monitor the polarity of the environment and determine the critical micelle concentration based on the ratio of the intensity of the pyrene monomer emission of the first (I_1_ at 373 nm) and third peak (I_3_ at 384 nm) [31,32,33].

In this paper, we prepared a series of pyrenyl-containing PDMAA copolymers by free radical copolymerization of DMAA with pyrenebutanoyloxy ethyl methacrylate (PyBEMA). The structure of the as-prepared copolymers was confirmed through UV, GPC, FT-IR and ^1^H NMR spectroscopy. We then investigated the effect of cyclodextrins (α-CD, β-CD and γ-CD) on thermosensitivity and fluorescence of the copolymers in aqueous solutions.

## 2. Materials and Methods

### 2.1. Materials

*N*,*N*-dimethylacrylamide (DMAA, 99%), α-cyclodextrins (α-CD, 98%), β-cyclodextrins (β-CD, 98%), γ-cyclodextrins (γ-CD, 98%) and 1-pyrenebutyric acid (PyBA, 97%) were purchased from J&K Scientific Co., Ltd. (Shanghai, China). Azobisisobutyronitrile (AIBN) was purchased from Tianjin Bodi Chemical Co., Ltd. (Tianjin, China) and purified by recrystallization from ethanol. 2-Hydroxyethyl methacrylate (HEMA, 99%) was purchased from Shanghai Aladdin Chemical Reagent Co., Ltd. (Shanghai, China). Dicyclohexylcarbodiimide (DCC) and 4-dimethylaminopyridine (DMAP) were purchased from Sinopharm Chemical Reagent Co., Ltd. (Beijing, China). Tetrahydrofuran (THF), dichloromethane, diethyl ether and ethanol were used as received from commercial sources. All other reagents were used without further purification.

### 2.2. Preparation of 2-(1-Pyrenebutanoyloxy)ethyl Methacrylate (PyBEMA)

2-(1-Pyrenebutanoyloxy)ethyl methacrylate (PyBEMA) was prepared by room condensation of HEMA and PyBA in presence of DCC and DMAP. In brief, PyBA (2.88 g, 10 mmol) was dissolved in 30 mL of THF in a 100 mL flask, to which DCC (2.16 g, 10.5 mmol) and DMAP (244 mg, 2 mmol) were added. After stirring for 20 min, HEMA (1.95 g, 15 mmol) was added into above mixture. After stirring for 24 h at room temperature, the resulting precipitate was filtered off and the solution was concentrated by rotary evaporation. The crude product was recrystallized from dichloromethane/ethanol mixture and dried in vacuum at 40 °C for 24 h. Pure PyBEMA was obtained with the yield of 68% as a yellow solid, m.p. 67–69 °C. FT-IR (KBr, cm^−1^): 1750, 1710 (s, C = O), 1630, 1610, 1530 (m, pyrene ring), 1160 (s, C-O), 950 (m, C = CH_2_), 842 (s, pyrene-H). ^1^H NMR (400 MHz, CDCl_3_, δ in ppm): 8.35–7.82 (m, 9 H, pyrene-H), 6.11 (s, 1 H, C = CH_2_), 5.54 (s, 1 H, C = CH_2_), 4.36 (s, 4 H, OCH_2_CH_2_O), 3.40 (m, 2 H, Pyrene-CH_2_), 2.49 (*t*, 2 H, Pyrene-CH_2_CH_2_CH_2_), 2.21 (m, 2 H, Pyrene-CH_2_CH_2_CH_2_), 1.91 (s, 3 H, CH_3_). UV (ethanol): λ = 343 nm, 327 nm, 313 nm, 276 nm, 265.5 nm, 255 nm, 243 nm, 234 nm.

### 2.3. Preparation of Pyrenyl-Containing PDMAA Copolymers

**Scheme 1:**
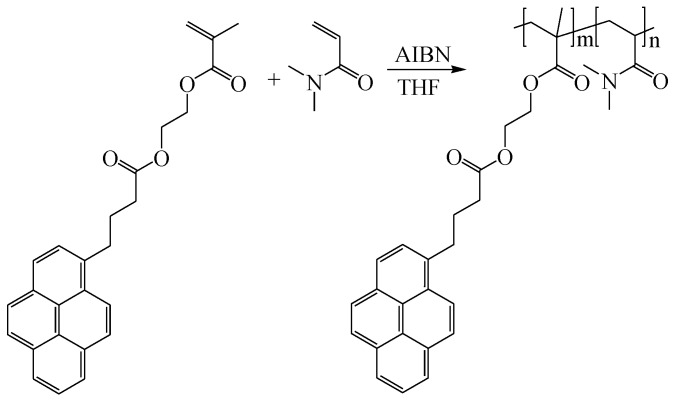
Synthesis of pyrenyl-containing PDMAA copolymers.

A series of pyrenyl-containing PDMAA copolymers (structure as shown in Scheme 1) were prepared by free radical copolymerization of DMAA with PyBEMA in THF (conc. of monomer 1 mol/L) using AIBN (0.3 mol %) as initiator at 60 °C for 16 h (Table 1). The polymer was purified by precipitating from THF into diethyl ether three times, and dried in a vacuum at 40 °C for 24 h to yield a yellow solid.

### 2.4. Characterization and Measurements

UV-Vis spectra were measured on a PerkinElmer Lambda 365 UV-Vis spectrometer using a 1 cm path length quartz cuvette. FT-IR spectra were recorded on a Nicolet iS50 FT-IR spectrometer (Nicolet, Waltham, MA, USA) using KBr pellets. ^1^H NMR spectra were performed on a Bruker AVANCE AV 400 NMR spectrometer (Bruker, Basel, Switzerland). Thermogravimetric analysis (TGA) was performed on a TA Q600 (TA Instruments, New Castle, DE, USA) at a scan rate of 10 °C·min^−1^ under nitrogen atmosphere. Gel permeation chromatography (GPC) measurements were performed on a Waters 1525 system equipped with a HT4 styragel column (40 °C) and Waters 2414 detectors (35 °C). THF was used as an eluent with an elution rate of 1.0 mL·min^−1^. The molecular weights were calibrated with polystyrene standards. Steady-state fluorescence emission spectra were recorded on a HORIBA Jobin Yvon FluoroMax 4 spectrometer (Horiba Jobin Yvon Inc., Edison, NJ, USA) equipped with TZL-1006D low constant temperature water baths. Cloud point (T_cp_), defined as the temperature for the change half of transmittance, was determined by measuring the transmittance of 0.5% aqueous solution sample at 600 nm with the heating rate of 0.5 °C·min^−1^ (Lambda 750s UV-Vis-NIR spectrometer equipped with a PTP-1+1 Peltier heated pool rack).

## 3. Results and Discussion

### 3.1. Preparation and Characterization of Pyrenyl-Containing PDMAA Copolymers

First, a series of pyrenyl-containing PDMAA copolymers were prepared by free radical copolymerization of DMAA with PyBEMA varying the monomer feed ratios (Scheme 1). The pyrenyl content in the copolymers, referred as molar ratio of PyBEMA unit versus DMAA unit in the copolymers, was estimated by UV-Vis absorption of pyrenyl group, assuming that there was the same absorption coefficient of pyrenyl group in the PyBEMA monomer and its copolymer at band of 342 nm. Also it was calculated by ^1^H NMR spectroscopy of the copolymers. The molecular weight and its distribution were determined by GPC based on polystyrene standards using THF as an eluent. The results of copolymerization (Table 1) show that the content of the pyrenyl group in the copolymers is less than that in the feed except for PDMAA-5.7 determined by ^1^H NMR, which probably resulted from different monomer reactivity ratios. Molar content of the pyrenyl group determined by UV spectra is basically consistent with that calculated by ^1^H NMR. The yield of copolymer increases with the increasing feed ratio of PyBEMA as a result of poorer solubility of higher pyrenyl content copolymer in diethyl ether precipitant. The average molecular weight (M_n_) of the as-synthesized copolymers is not high and the polydispersity index (PDI, M_w_/M_n_) of relative molecular weight increases with the increasing feed ratio of PyBEMA since the pyrenyl monomer appears to act as a chaintransfer agent during the polymerization [12].

**Figure 1:**
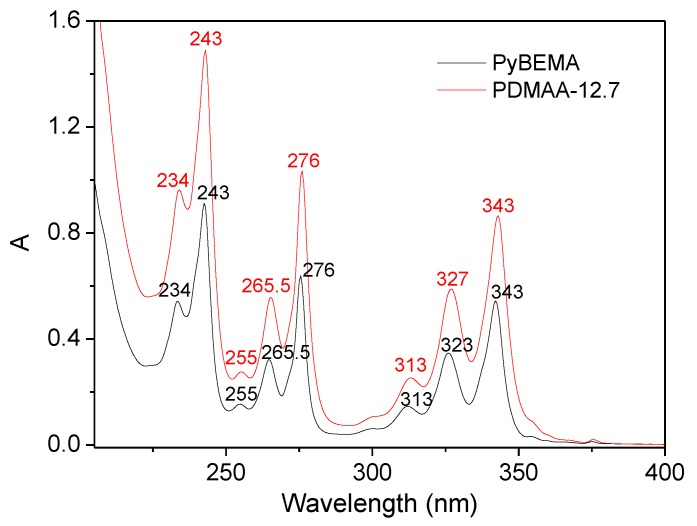
UV absorption spectra of PyBEMA monomer and PDMAA-12.7 copolymer in ethanol.

**Figure 2:**
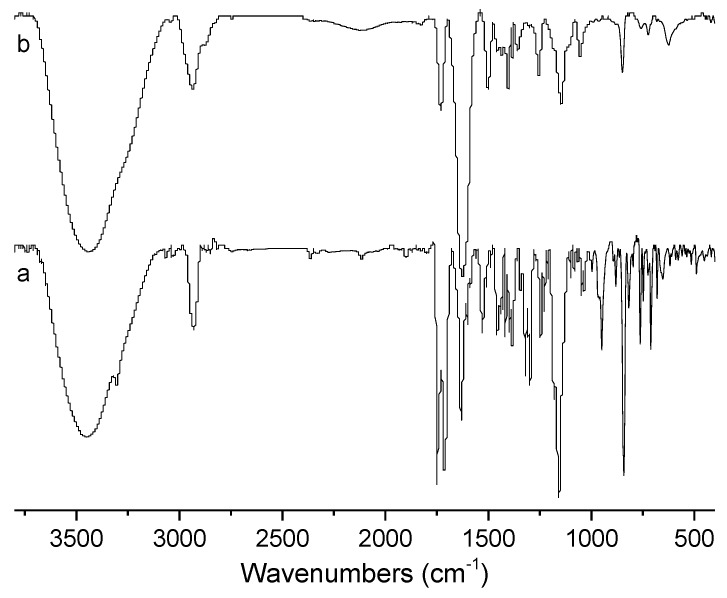
FT-IR spectra of PyBEMA monomer (**a**) and PDMAA-12.7 copolymer (**b**).

**Figure 3:**
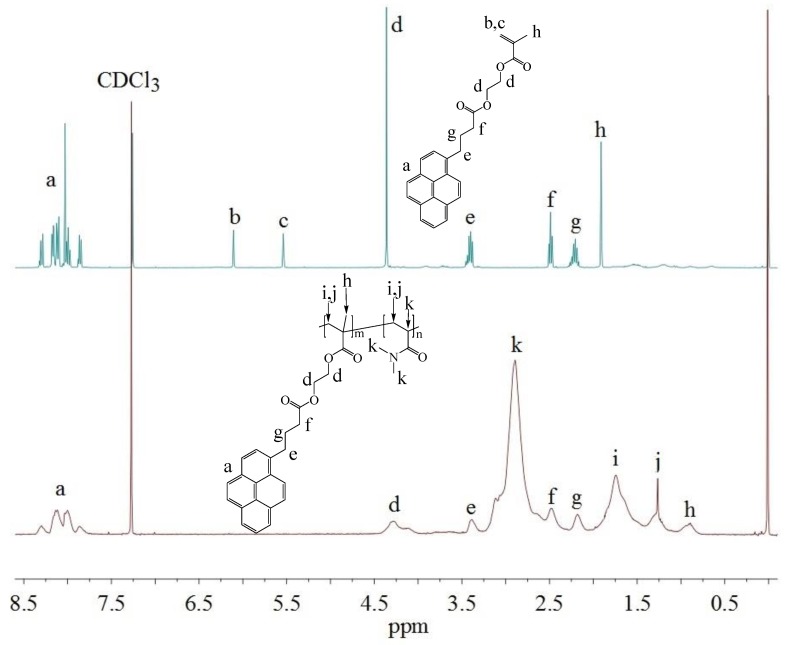
^1^H NMR spectra of PyBEMA monomer and PDMAA-12.7 copolymer in CDCl_3_.

Secondly, the chemical structure of the as-synthesized pyrenyl-containing PDMAA copolymers was characterized by UV, FT-IR and ^1^H NMR spectroscopy. Figure 1 shows UV absorption spectra of PyBEMA monomer and PDMAA-12.7 copolymer in ethanol. The UV absorption spectrum of pyrenyl moiety exhibits the same characteristic absorption bands nearby 343 nm, 276 nm and 243 nm, respectively, whether in monomer or copolymer, which is consistent with that of pyrene reported by Ray and co-workers [34]. Compared with that of PyBEMA monomer (Figure 2), FT-IR spectrum of PDMAA-12.7 copolymer shows absorption band at about 1730 cm^−1^ attributed to the C = O stretching vibration of the α,β-saturated ester group originating from PyBEMA units. The strong absorption band at about 1620 cm^−1^ is attributed to the C = O stretching vibrations of a secondary amide. The characteristic peaks of pyrenyl skeleton vibration in the copolymer is overlapped by that of the amide group, which should appear in 1630 cm^−1^ and 1610 cm^−1^. The band at 849 cm^−1^ is assigned to C-H bending vibration of pyrenyl ring. The absorption bands in 1500 cm^−1^ and 1450 cm^−1^ are assigned to C-H bending vibration of *N*,*N*-dimethyl group. The band in 1145 cm^−1^ belongs to the C-O stretching vibration from PyBEMA units. After polymerization, the bands at 1710 cm^−1^ attributed to the C = O stretching vibration of the α,β-unsaturated ester group and 950 cm^−1^ attributed to C-H bending vibration of vinyl group in PyBEMA monomer disappear in the copolymer. Furthermore, ^1^H-NMR spectra confirmed the pyrenyl group in the copolymer (Figure 3). The resonance peaks 7.7–8.4 ppm belong to the protons of the pyrenyl group. The resonance bands at 4.3 ppm and at 3.4 ppm, 2.5 ppm and 2.2 ppm are assigned to the –OCH_2_CH_2_O- and -CH_2_CH_2_CH_2_C = O groups in the side chain from PyBEMA units, respectively. The resonance peaks in the region 2.6-3.2 ppm are assigned to the *N*,*N*-dimethyl group in the side chain and the -CH-C = O group in the main chain. The bands from 1.1 ppm to 2.0 ppm are assigned to the -CH_2_- group in the main chain. The resonance peak 0.9 ppm belongs to the protons of the -CH_3_ group from PyBEMA units. The sharp line at 1.25 ppm originates from the AIBN initiator debris attached to the end of the polymer. The proton integrated signals between 5.4 ppm to 6.8 ppm from -CH_2_ = CH- and -CH_2_ = C- in DMAA and PyBEMA disappear in PDMAA-12.7 copolymer.

### 3.2. Effect of Cyclodextrins on Thermosensitivity of Pyrenyl-Containing PDMAA Copolymers

**Figure 4:**
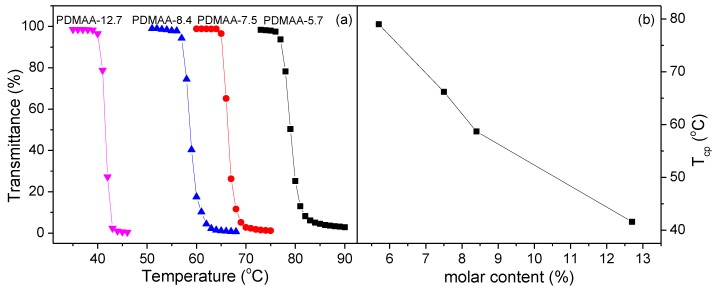
Transmittance as function of temperature (**a**) and T_cp_ related to molar content of PyBEMA in copolymer (**b**) for 5 g/L pyrenyl-containing PDMAA copolymers in aqueous solution.

Thermosensitivity of the as-synthesized pyrenyl-containing PDMAA copolymers in aqueous solution was investigated by determining temperature-dependent transmittance. Cloud point (T_cp_) was used to characterize the phase separation temperature of the as-synthesized copolymers. As shown in Figure 4, pyrenyl-containing PDMAA copolymers in aqueous solutions exhibit LCST-type thermosensitivity. T_cp_ decreases with the increasing molar content of PyBEMA units in the copolymers, which is the result of the increasing of the hydrophobic pyrenyl group.

Furthermore, the effect of types and quantity of cyclodextrins (α-CD, β-CD and γ-CD) on the thermosensitivity of pyrenyl-containing PDMAA copolymers in aqueous solution was investigated, as shown in Table 2 and Figures S2–S13 (Supporting Information). It is well known that the CD is water-soluble, has different cavity size for α-CD, β-CD and γ-CD, and is capable of selectively including a wide range of hydrophobic guest molecules [35,36,37]. Pyrenyl-containing PDMAA copolymers still have LCST-type thermosensitivity after the addition of the CD no matter which CD is added (see Supporting Information Figures S1–S12). T_cp_ of pyrenyl-containing PDMAA copolymers increases after the CD is added from half molar to equivalent amount relative to pyrenyl moiety, which is the result of an increase in polymer hydrophilicity by the formation of 1:1 inclusion complexes between the CD molecule and pyrenyl moiety in the side chain of polymers. However, further adding twice equivalent CD results in a slight decrease in T_cp_, probably owing to dehydration of excessive CD after the formation of 1:1 inclusion complexes. Additionally, it is found that the increment of T_cp_ after the addition of the CD gradually becomes larger with a higher number of PyBEMA units in the copolymers, which may be explained by more changes from hydrophobic pyrenyl groups to hydrophilic inclusion complex with the higher number of PyBEMA units. Compared with the three kinds of CD, the effect of γ-CD on the increase of T_cp_ is not as obvious as that of α-CD and β-CD, most likely due to instability of formed inclusion complexes between γ-CD and pyrenyl moiety.

### 3.3. Effect of Cyclodextrins on Fluorescence of Pyrenyl-Containing PDMAA Copolymers

**Figure 5:**
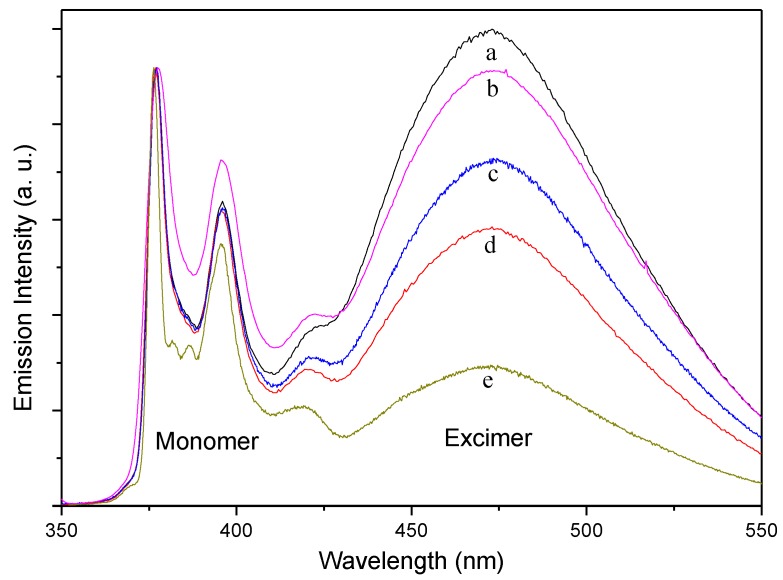
Fluorescence spectra of 10 μg/L PDMAA-12.7 copolymers (**a**) in water, (**b**) in water with equivalent γ-CD, (**c**) in water with equivalent β-CD, (**d**) in water with equivalent α-CD and (**e**) in ethanol. Excitation wavelength was 344 nm and the emission spectra were normalized at 377 nm.

Firstly, fluorescence spectra of 10 μg/L PDMAA-12.7 copolymers were measured in ethanol and in water with or without equivalent α-CD, β-CD and γ-CD, respectively. As shown in Figure 5, the copolymers exhibit an emission located at 377 nm (intensity I_M_) and a broad emission centered at 470 nm (intensity I_E_), whether in ethanol or water with or without α-CD, β-CD and γ-CD, which are the result of locally excited pyrene monomer chromophores with the [0,0] band and pyrene excimer chromophores emission. Similar excitation spectra were obtained for emission wavelength fixed nearby at 472 nm and 377 nm, and the peak maxima and shape correspond to those in the UV absorption spectra (see Supporting Information Figures S14–S18). The excitation spectra for the monomer are blue-shifted by about 4 nm compared with that for the excimer, which correspond to the report by Winnik [12]. The fluorescence spectrum of the copolymers in water shows a stronger excimer emission relative to monomer emission than that in ethanol, which is probably explained by increasing pairs of aggregates of pyrenes due to hydrophobic association of the copolymers in water. The values of I_E_/I_M_ decrease after the addition of equivalent α-CD, β-CD and γ-CD into the copolymers in aqueous solution respectively, originating from the isolation effect by the formation of 1:1 inclusion complex between pyrene moiety and the CD through host-guest molecular recognition. The excimer emission remains strong after adding γ-CD for the instability of the formed inclusion complex between γ-CD and pyrenyl moiety.

**Figure 6:**
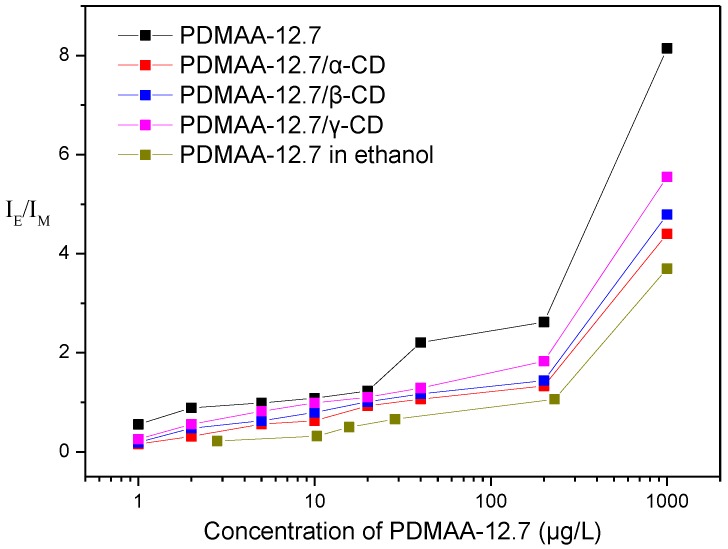
The ratio of excimer to monomer emission intensities (I_E_/I_M_) as a function of the concentration of PDMAA-12.7 copolymers in ethanol and in water with equivalent α-CD, β-CD and γ-CD, respectively.

Secondly, the ratio of I_E_/I_M_ were determined for solutions of PDMAA-12.7 copolymers in ethanol and in water with equivalent α-CD, β-CD, and γ-CD, respectively, as a function of the concentration. As shown in Figure 6, the values I_E_/I_M_ increase slightly with the increase in the copolymers concentration when the concentration is below 0.2 mg/L. The sharp increase takes place for solutions of concentration over 0.2 mg/L. From the above results, it can be inferred that intra-polymeric pyrene aggregates dominate for solution concentrations below 0.2 mg/L and inter-polymeric pyrene aggregates dominate for solution concentrations over 0.2 mg/L. The same trends were observed whether in ethanol solution or aqueous solution with or without α-CD, β-CD, and γ-CD, respectively. Normally, the formation of inclusion complexes after the addition of the CD will hinder intra- and inter-polymeric pyrene aggregates. However, it still forms pyrene aggregates, probably as the formation of the CD pseudopolyrotaxanes in the side chain make it possible by putting CD into the spacer.

**Figure 7:**
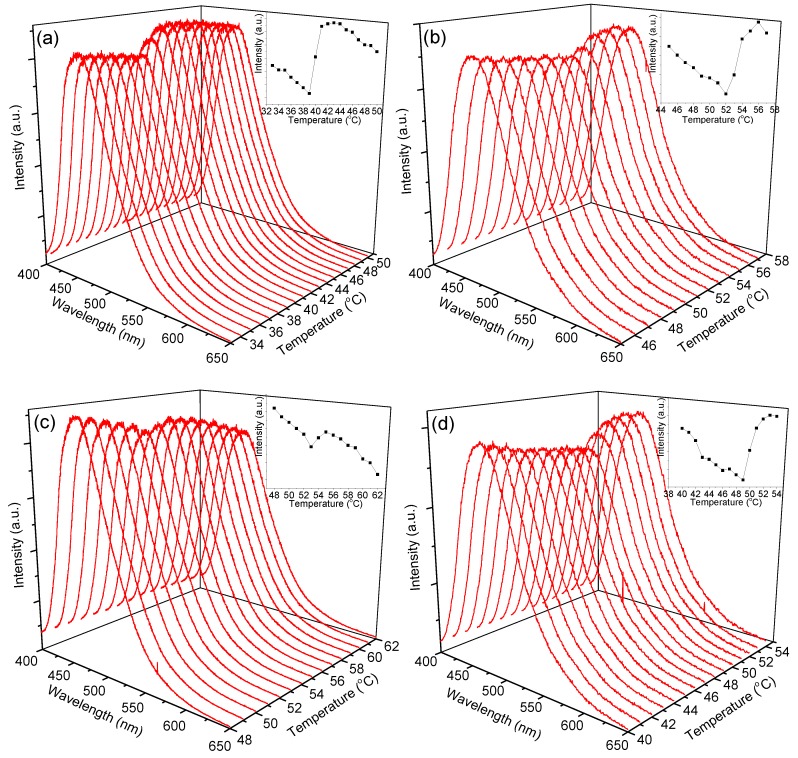
Excimer fluorescent spectra of 5 g/L PDMAA-12.7 copolymers in aqueous solution as a function of temperature (**a**) in the absence of the CD, (**b**) in the presence of equivalent α-CD, (**c**) in the presence of equivalent β-CD and (**d**) in the presence of equivalent γ-CD. Inserts are the curve of the maximum emission intensity related to temperature.

Finally, temperature sensitive fluorescent spectra of 5 g/L PDMAA-12.7 copolymers in aqueous solution were measured in the absence of the CD and in the presence of equivalent α-CD, β-CD and γ-CD, respectively (Figure 7). Here, the concentration for fluorescence determination is up to 4.2 × 10^−3^ mol/L and quite high for pyrene chromophores. As a result, the copolymers completely exhibit an excimer emission located at about 370 nm. As the temperature increased, all the characteristic excimer emission peaks were almost unchanged. However, the values I_E_ abruptly increased as the temperature went up to some point close to T_cp_. Generally, the fluorescence intensity decreases with the increase of temperature because of the more enhanced nonradiative decay in higher temperature. As the phase separation of the copolymers takes place, the hydrated shell around the pyrene excimer fluorophore changes to hydrophobic polymer chains, greatly weakening the interaction of the excitated excimer with nearby molecules and resulting in the abrupt increase of I_E_. Furthermore, it was found that the temperature for abrupt change of I_E_ was about 1 K below cloud point. This phenomenon may be due to the microscopic hydrophobic association detected by fluorescence having already taken place as the macroscopic cloud point is observed with the increasing temperature. The addition of the CD does not change excimer emission peaks and the trend of intensity with respect to the change of temperature. The formation of the CD pseudopolyrotaxanes leads to the change of the phase separation temperature of the polymer solutions but hardly affects the abrupt change of I_E_ around cloud point. In another way, the values I_E_ also can be used to research phase separation of polymers.

## 4. Conclusions

In summary, we have reported the preparation of pyrenyl-containing PDMAA copolymers and investigated the effect of cyclodextrins (α-CD, β-CD and γ-CD) on thermosensitivity and fluorescence of pyrenyl-containing PDMAA copolymers in aqueous solution. Pyrenyl-containing PDMAA copolymers in aqueous solutions exhibit LCST-type thermosensitivity and T_cp_ decreases with the increasing molar content of PyBEMA units in the copolymers. The addition of cyclodextrins (CD) such as α-CD, β-CD and γ-CD, does not change the LCST-type temperature-stimuli sensitivity of the copolymers. T_cp_ of the copolymers increases after the addition of the CD due to the formation of 1:1 inclusion complex between pyrenyl side chain and the CD. Excessive CD causes the dehydration of the hydrated copolymers and a slight decrease in T_cp_. The increment of T_cp_ after the addition of the CD gradually become larger in the copolymers with higher numbers of PyBEMA units. The cavity size of γ-CD does not exactly match the pyrenyl group which results in the instability of the formed inclusion complex between γ-CD and pyrenyl moiety. So, the effect of γ-CD on the increase of T_cp_ is not as obvious as that of α-CD and β-CD. The copolymers exhibit a pyrene emission located at 377 nm and a broad excimer emission centered at 470 nm. The fluorescence spectrum of the copolymers in water shows a stronger excimer emission relative to monomer emission than that in ethanol, resulting from increasing pairs of aggregates of pyrenes due to hydrophobic association of the copolymers in water. The I_E_/I_M_ values decrease after the addition of equivalent α-CD, β-CD and γ-CD into the copolymers in aqueous solution, respectively. The I_E_/I_M_ values as a function of concentration indicate that intra-polymeric pyrene aggregates dominate for solution concentrations below 0.2 mg/L and that inter-polymeric pyrene aggregates dominate for concentrations over 0.2 mg/L. The formation of the CD pseudopolyrotaxanes makes it possible to form pyrene aggregates at various concentrations. For high concentration of 5 g/L, the copolymers and their inclusion complexes completely exhibit an excimer emission. The I_E_ values abruptly increase as the temperature rises to T_cp_, which indicates that the I_E_ values can be used to research phase separation of polymers.

## Figures and Tables

**Table 1:** Copolymerization of dimethylacrylamide (DMAA) with pyrenebutanoyloxy ethyl methacrylate (PyBEMA).

Sample Code	Molar Feed Ratio of PyBEMA (%) ^1^	Molar Content of PyBEMA in Copolymer (%)		Yield (%)	Molecular Weight ^4^	
		UV ^2^	^1^H NMR ^3^		M_n_	M_w_/M_n_
PDMAA-5.7	6	5.7	6.1	52	5400	1.67
PDMAA-7.5	10	7.5	7.1	60	5500	1.76
PDMAA-8.4	12	8.4	8.3	62	5800	1.79
PDMAA-12.7	14	12.7	12.4	70	4700	2.05

^1^ Referred as molar ratio of PyBEMA versus DMAA. ^2^ Referred as molar ratio of PyBEMA unit versus DMAA unit in the copolymers. ^2^ Determined by UV-Vis spectroscopy based on the same absorption coefficient of pyrenyl group in PyBEMA monomer and copolymer at band of 343 nm. ^3^ Calculated by ^1^H NMR spectroscopy (See Supporting Information). ^4^ Determined by GPC based on PS standards in THF.

**Table 2:** T_cp_ of copolymer in aqueous solution related types and quantity of cyclodextrins.

Sample Code	No Cyclodextrins	α-Cyclodextrins			β-Cyclodextrins			γ-Cyclodextrins		
		0.5	1	2	0.5	1	2	0.5	1	2
PDMAA-5.7	79.0	84.9	87.7	87.3	85.0	88.8	87.9	81.6	82.8	81.6
PDMAA-7.5	66.2	72.7	76.8	75.6	73.6	77.6	76.0	70.4	72.7	72.0
PDMAA-8.4	58.7	65.9	70.4	69.5	64.5	69.6	68.7	63.7	65.7	65.1
PDMAA-12.7	41.6	48.7	54.7	52.9	49.5	55.9	53.5	47.6	51.1	49.6

## Supplementary Materials

The following are available online at https://www.mdpi.com/2073-4360/11/10/1569/s1, Figures S1–S18, details theoretical calculation of pyrene molecule size.
