# Supplementary material for: Influence of Cyclodextrins on Thermosensitive and Fluorescent Properties of Pyrenyl-Containing PDMAA
Source: Polymers (Basel). 2019 Sep 26;11(10):1569. doi: 10.3390/polym11101569 (PMC6835872; doi:10.3390/polym11101569)
Supplement: Supplementary file 1 [file polymers-11-01569-s001.pdf]

polymers

Supplementary Materials

Influence of Cyclodextrins on Thermosensitive and Fluorescent Properties of Pyrenyl-Containing PDMAA

Qiujing Dong [1,2,3], Changrui Sun [1,2], Fangyuan Chen [1,2], Zheng Yang [1,2], Ruiqian Li [1,2], Chang Wang [1,2] and Chunhua Luo [1,2,*]

1 School of Chemistry and Materials Engineering, Fuyang Normal University, Fuyang 236037, China; dongqj1980@163.com (Q.D.); sunchangrui1208@163.com (C.S.); cfy8861@qq.com (F.C.); zhengyang8402@qq.com (Z.Y.); liruiqian2008@163.com (R.L.); bigceleron@163.com (C.W.)
2 Anhui Provincial Key Laboratory for Degradation and Monitoring of the Pollution of the Environment, Fuyang 236037, China
3 State Key Laboratory of Molecular Engineering of Polymers (Fudan University), Shanghai 200433, China
* Correspondence: ch-luo@fynu.edu.cn; lch197919@163.com; Tel.: +86-558-259-6249

Received: 31 August 2019; Accepted: 23 September 2019; Published: date

1. *Calculation of molar content of PyBEMA in copolymer by ^{1}H NMR*

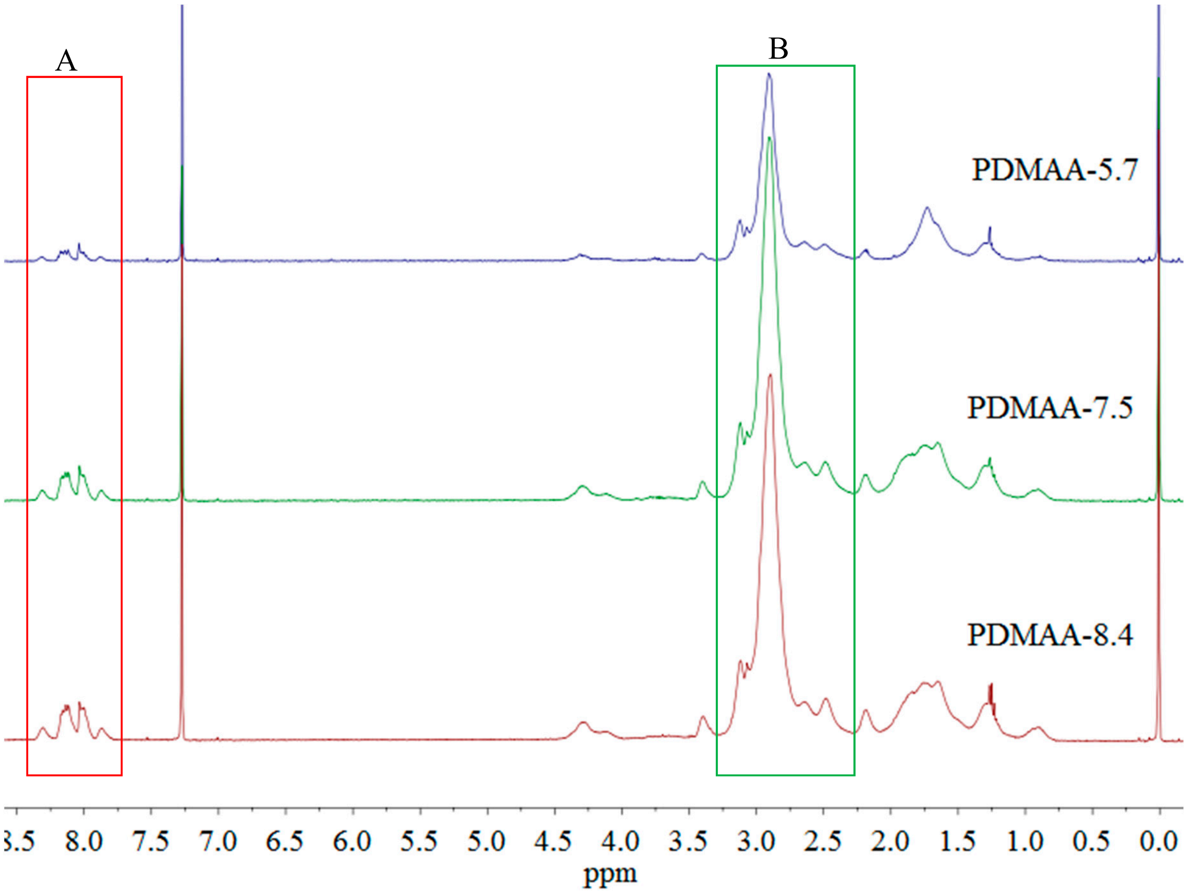

Figure S1. ^{1}H NMR spectra of PDMAA-5.7, PDMAA-7.5 and PDMAA-8.4 in $CDCl_3$.

"A" represents protons integral area of pyrene ring (a) and "B" represents protons integral area of N,N-dimethyl (k), CH-C=O (k) and CH_2 (f). Molar content of PyBEMA in copolymer x% is calculated as follow.

$$x = \frac{\frac{A}{9}}{\frac{B - \frac{A \times 2}{9}}{7}} \times 100$$

	PDMAA-5.7	PDMAA-7.5	PDMAA-8.4	PDMAA-12.7
A	1	1	1	1
B	13.073	11.254	9.557	6.509
x	6.1	7.1	8.3	12.4

2. *Transmittance-Temperature Relationship Curves*

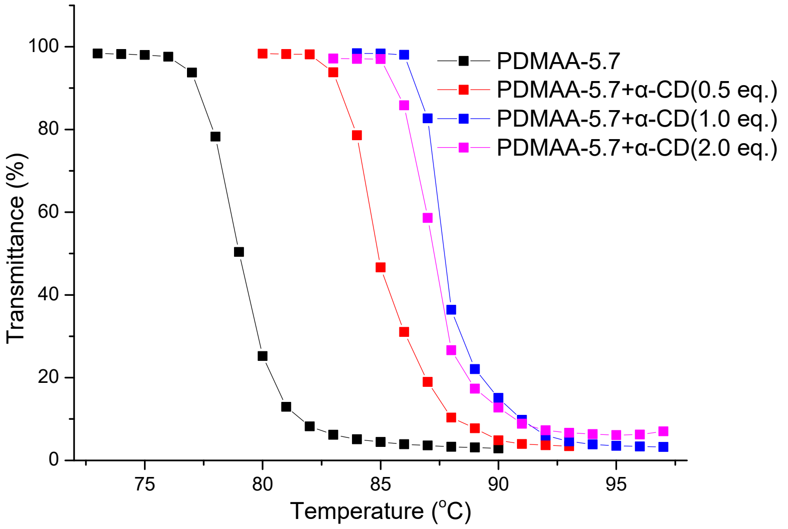

Figure S2. Transmittance as function of temperature for 5 g/L PDMAA-5.7 copolymers aqueous solution in the presence of different amount of α-CD.

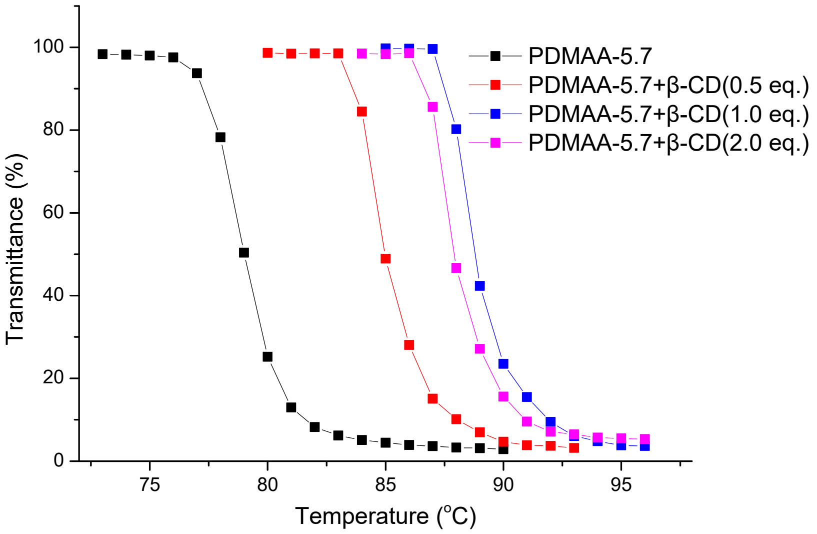

Figure S3. Transmittance as function of temperature for 5 g/L PDMAA-5.7 copolymers aqueous solution in the presence of different amount of β-CD.

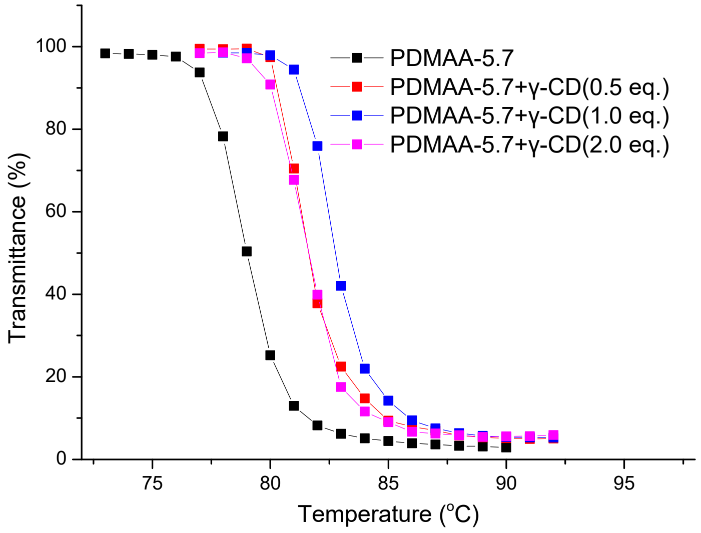

Figure S4. Transmittance as function of temperature for 5 g/L PDMAA-5.7 copolymers aqueous solution in the presence of different amount of γ-CD.

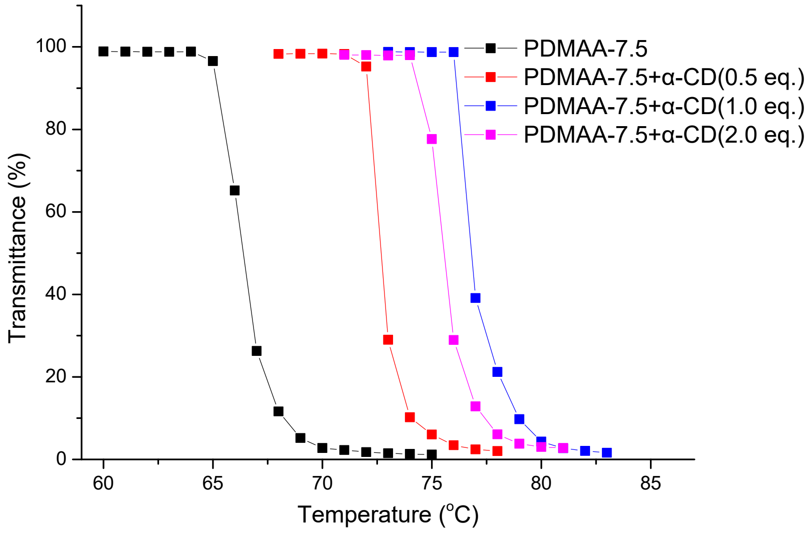

Figure S5. Transmittance as function of temperature for 5 g/L PDMAA-7.5 copolymers aqueous solution in the presence of different amount of α-CD.

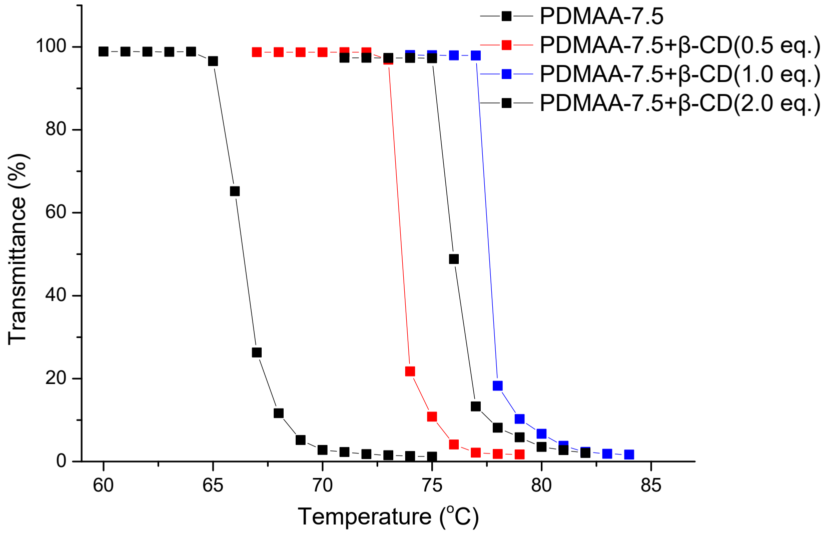

Figure S6. Transmittance as function of temperature for 5 g/L PDMAA-7.5 copolymers aqueous solution in the presence of different amount of β-CD.

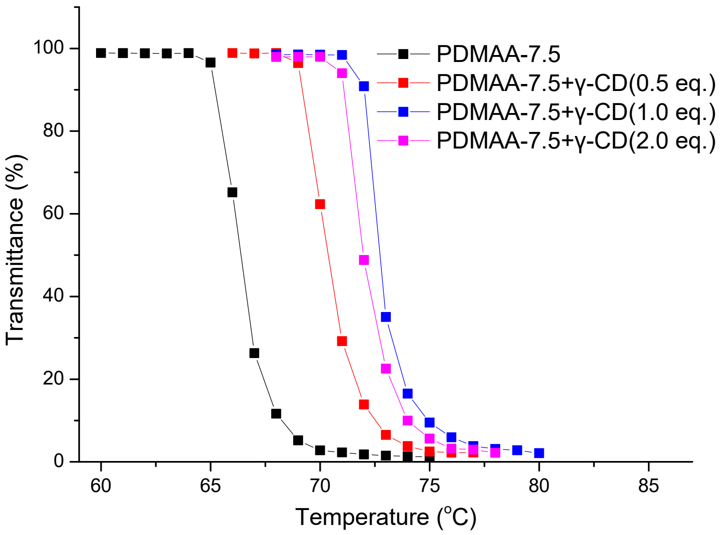

Figure S7. Transmittance as function of temperature for 5 g/L PDMAA-7.5 copolymers aqueous solution in the presence of different amount of γ-CD.

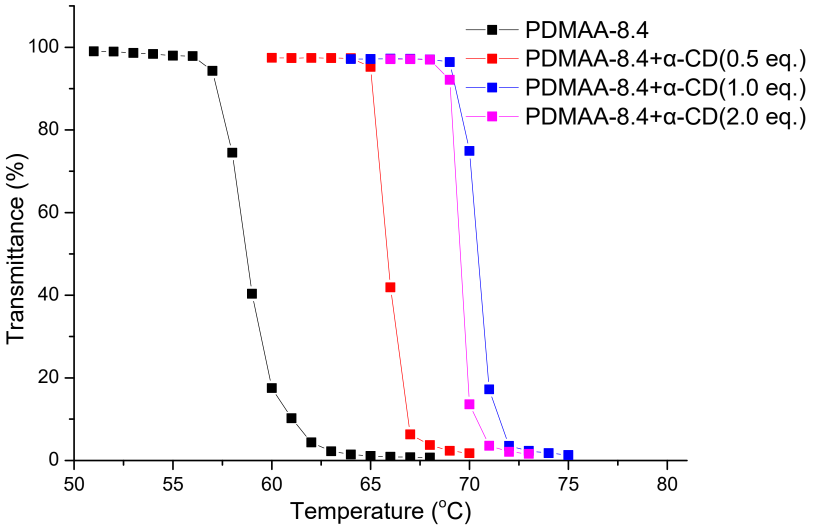

Figure S8. Transmittance as function of temperature for 5 g/L PDMAA-8.4 copolymers aqueous solution in the presence of different amount of α-CD.

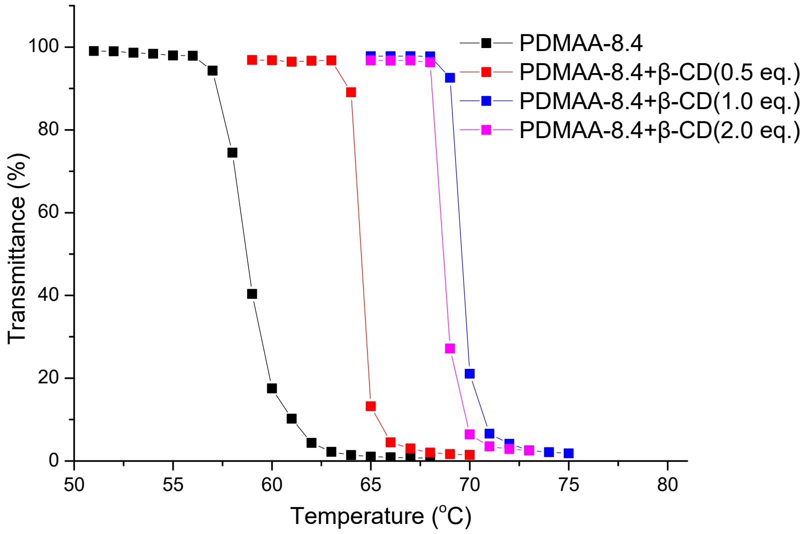

Figure S9. Transmittance as function of temperature for 5 g/L PDMAA-8.4 copolymers aqueous solution in the presence of different amount of β-CD.

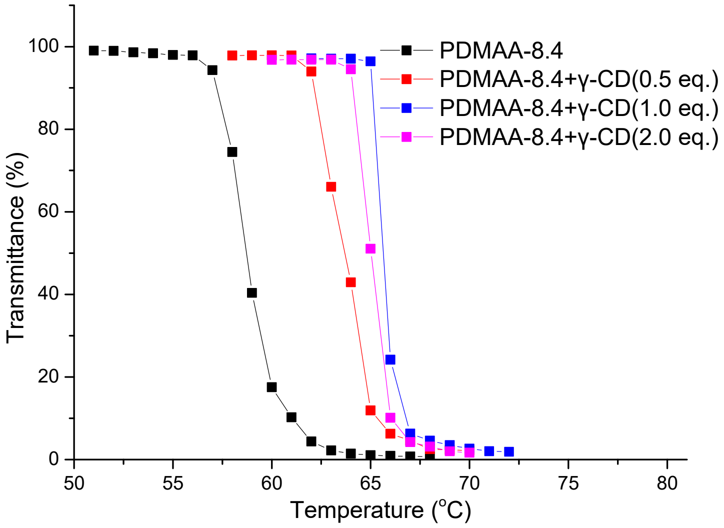

Figure S10. Transmittance as function of temperature for 5 g/L PDMAA-8.4 copolymers aqueous solution in the presence of different amount of γ-CD.

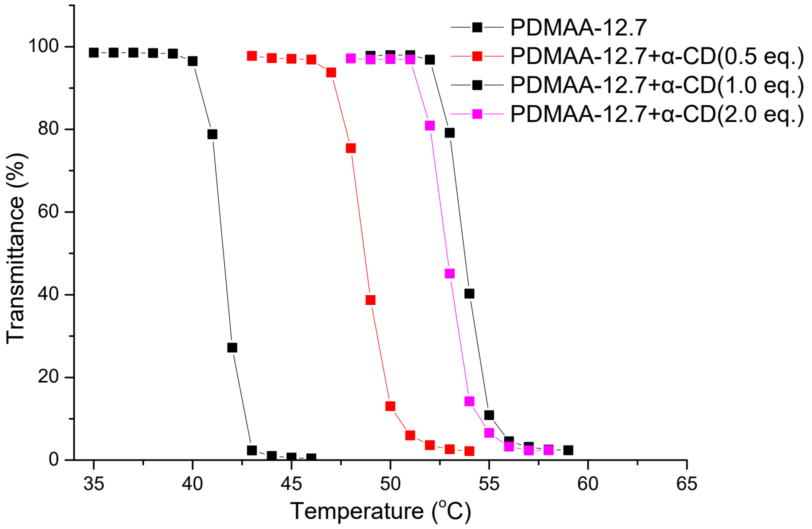

Figure S11. Transmittance as function of temperature for 5 g/L PDMAA-12.7 copolymers aqueous solution in the presence of different amount of α-CD.

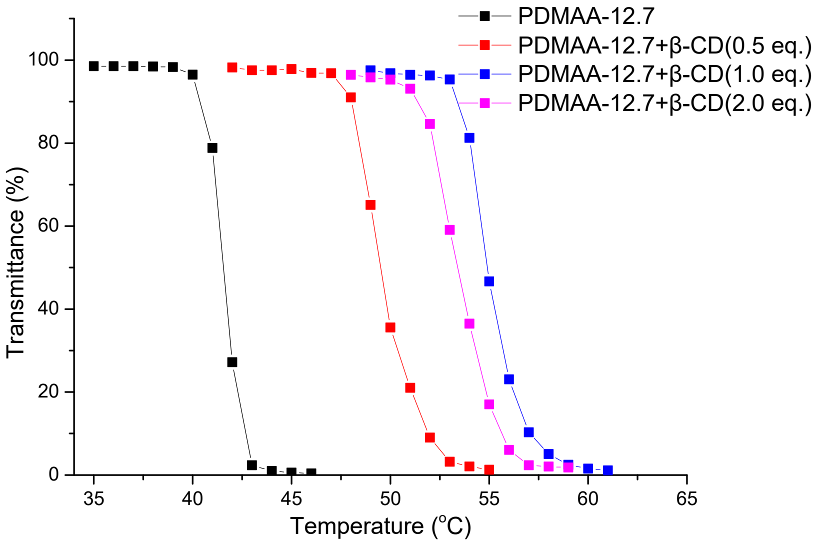

Figure S12. Transmittance as function of temperature for 5 g/L PDMAA-12.7 copolymers aqueous solution in the presence of different amount of β-CD.

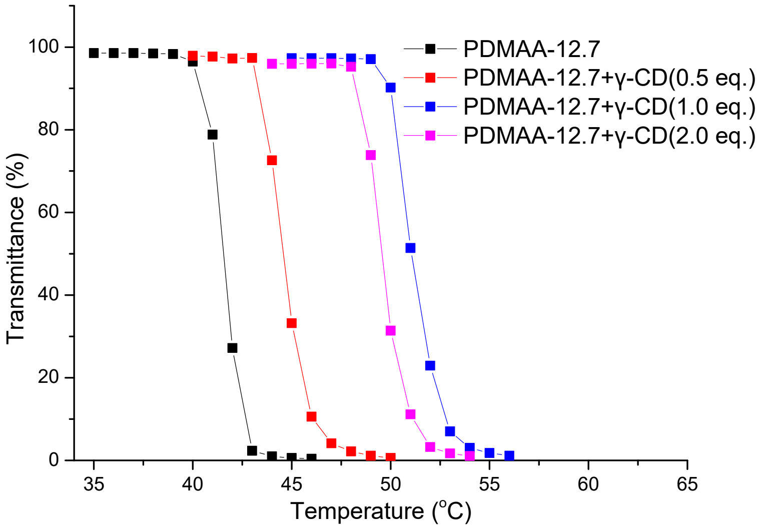

Figure S13. Transmittance as function of temperature for 5 g/L PDMAA-12.7 copolymers aqueous solution in the presence of different amount of γ-CD.

3. Excitation Spectra of 10 μg/L PDMAA-12.7 Copolymers

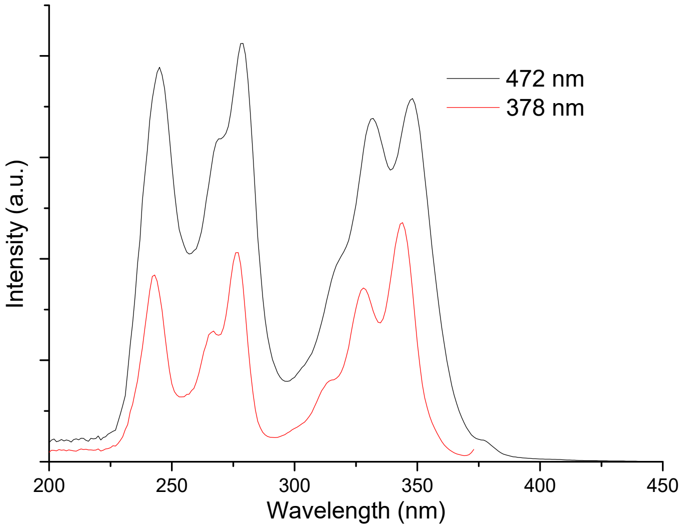

Figure S14. Excitation spectra of 10 μg/L PDMAA-12.7 copolymers aqueous solution. Emission wavelength was fixed at 472 nm (black curve) and 378 nm (red curve) respectively.

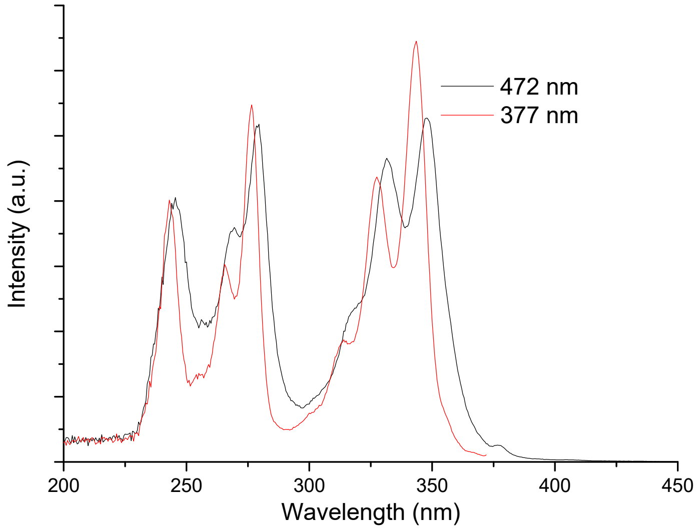

Figure S15. Excitation spectra of 10 μg/L PDMAA-12.7 copolymers aqueous solution with equivalent α-CD. Emission wavelength was fixed at 472 nm (black curve) and 377 nm (red curve) respectively.

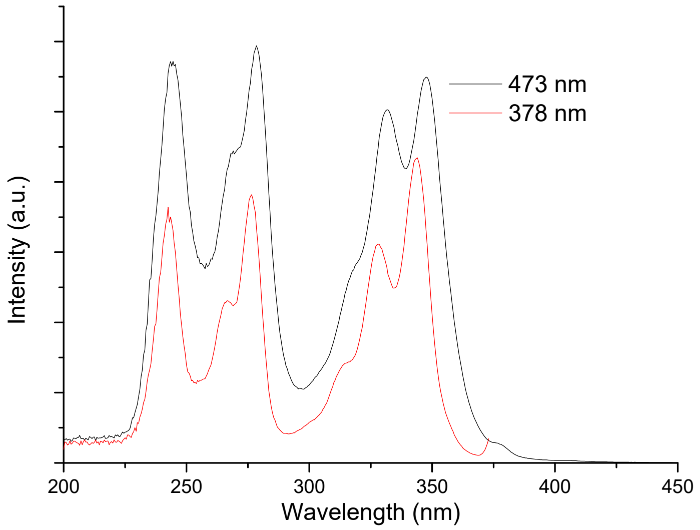

Figure S16. Excitation spectra of 10 μg/L PDMAA-12.7 copolymers aqueous solution with equivalent β-CD. Emission wavelength was fixed at 473 nm (black curve) and 378 nm (red curve) respectively.

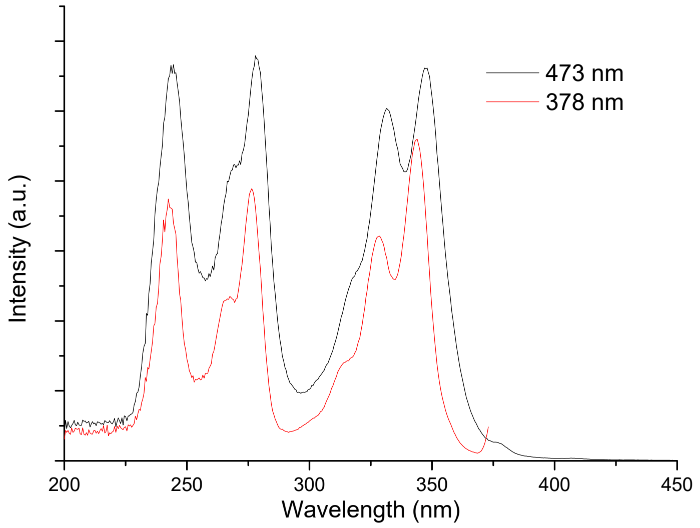

Figure S17. Excitation spectra of 10 μg/L PDMAA-12.7 copolymers aqueous solution with equivalent γ-CD. Emission wavelength was fixed at 473 nm (black curve) and 378 nm (red curve) respectively.

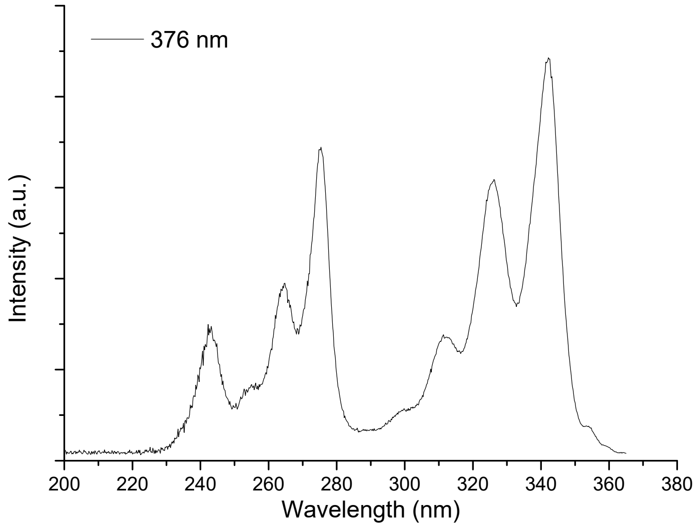

Figure S18. Excitation spectrum of 10 μg/L PDMAA-12.7 copolymers in ethanol. Emission wavelength was fixed at 376 nm.

© 2019 by the authors. Submitted for possible open access publication under the terms and conditions of the Creative Commons Attribution (CC BY) license (http://creativecommons.org/licenses/by/4.0/).
